# Surface expression of Cytokine Receptor-Like Factor 2 increases risk of relapse in pediatric acute lymphoblastic leukemia patients harboring *IKZF1* deletions

**DOI:** 10.18632/oncotarget.25411

**Published:** 2018-05-25

**Authors:** Agata Pastorczak, Lukasz Sedek, Marcin Braun, Joanna Madzio, Alicja Sonsala, Magdalena Twardoch, Wojciech Fendler, Karin Nebral, Joanna Taha, Marta Bielska, Patryk Gorniak, Magdalena Romiszewska, Michal Matysiak, Katarzyna Derwich, Monika Lejman, Jerzy Kowalczyk, Wanda Badowska, Maciej Niedzwiecki, Bernarda Kazanowska, Katarzyna Muszynska-Roslan, Grazyna Sobol-Milejska, Grazyna Karolczyk, Andrzej Koltan, Tomasz Ociepa, Tomasz Szczepanski, Wojciech Młynarski

**Affiliations:** ^1^ Department of Pediatrics, Hematology, Oncology and Diabetology, Medical University of Łódź, Łódź, Poland; ^2^ Department of Pediatric Hematology and Oncology, Medical University of Silesia, Zabrze, Poland; ^3^ Postgraduate School of Molecular Medicine, Medical University of Warsaw, Warsaw, Poland; ^4^ Department of Pathology, Chair of Oncology, Medical University of Łódź, Łódź, Poland; ^5^ Department of Biostatistics and Translational Medicine, Medical University of Łódź, Łódź, Poland; ^6^ Children's Cancer Research Institute (CCRI), Vienna, Austria; ^7^ Institute of Hematology and Transfusion Medicine, Warsaw, Poland; ^8^ Department of Pediatrics, Oncology and Hematology, Medical University of Warsaw, Warsaw, Poland; ^9^ Department of Pediatric Hematology, Oncology, Transplantology, Medical University of Poznań, Poznań, Poland; ^10^ Department of Pediatric Hematology and Oncology, Medical University of Lublin, Lublin, Poland; ^11^ Department of Pediatric Hematology and Oncology, Children's Hospital in Olsztyn, Olsztyn, Poland; ^12^ Department of Pediatrics, Hematology, Oncology and Endocrinology, Medical University of Gdańsk, Gdańsk, Poland; ^13^ Department of Transplantology, Pediatric Oncology and Hematology, Medical University of Wrocław, Wrocław, Poland; ^14^ Department of Pediatric Oncology, Medical University, Bialystok, Poland; ^15^ Department of Pediatrics, Medical University of Silesia, Katowice, Poland; ^16^ Regional Specialistic Pediatric Hospital, Kielce, Poland; ^17^ Department of Pediatric Hematology and Oncology, Collegium Medicum in Bydgoszcz, Mikolaj Kopernik University, Bydgoszcz, Poland; ^18^ Department of Pediatrics, Hematology and Oncology, Pomeranian Medical University, Szczecin, Poland

**Keywords:** acute lymphoblastic leukemia, minimal residual disease, relapse, gene

## Abstract

We prospectively examined whether surface expression of Cytokine Receptor-Like Factor 2 (CRLF2) on leukemic blasts is associated with survival and induction treatment response in pediatric B-cell precursor acute lymphoblastic leukemia (BCP-ALL) patients. Flow cytometric analysis of bone marrow-derived leukemia cells revealed that 7.51% (29/286) of 386 pediatric BCP-ALL patients were CRLF2-positive (CRLF2pos) at diagnosis. The median minimal residual disease (MRD) was lower in CRLF2pos than CRLF2-negative (CRLF2neg) patients on day 15 (MRD15) after induction therapy [0.01% (0.001-0.42%) vs. 0.45% (0.05-3.50%); p=0.001]. By contrast, the MRD15 was higher in Ikaros family Zinc Finger Protein 1 (*IKZF1)*-deleted BCP-ALL patients than in BCP-ALL patients without *IKZF1* deletions [1.18% (0.06-12.0%) vs 0.33% (0.03-2.6%); p=0.003]. Subgroup analysis showed that MRD15 levels were lower in *IKZF1Δ*/CRLF2pos patients than in *IKZF1Δ*/CRLF2neg patients [0.1% (0.02-5.06%) vs. 2.9% (0.25-12%); p=0.005]. Furthermore, MRD15 levels were higher in *IKZF1*WT/CRLF2neg patients than in *IKZF1*WT/CRLF2pos patients [0.40% (0.04-2.7%) vs. 0.001% (0.001-0.01%)]. Despite the low MRD15 levels, *IKZF1*Δ/CRLF2pos patients showed poorer relapse-free survival (RFS) than other patient groups (p=0.003). These findings demonstrate that surface CRLF2 expression is associated with increased risk of relapse in pediatric BCP-ALL patients harboring *IKZF1* deletions.

## INTRODUCTION

Acute lymphoblastic leukemia (ALL) is the most common pediatric malignancy and is characterized by long-term survival rates (80-85%), especially in developed countries [1]. In the remaining 15-20% of pediatric ALL patients, relapse is the major cause of death [2–5]. High-resolution genome-wide profiling and sequencing studies demonstrate that the risk of ALL relapse is associated with specific biological features of the leukemic cells including gene mutations, copy number variations and gene fusions. Large-scale genetic profiling of Philadelphia chromosome (Ph)-like ALL or *BCR-ABL1*-like ALL (Ph-positive ALL without BCR-ABL1 fusion protein) shows large number of genetic alterations in the cytokine receptor and kinase-signaling pathway genes that contribute to its aggressive phenotype and frequent disease recurrence [6, 7].

Nearly 47% of *BCR-ABL1-*like ALL cases show rearrangements in the Cytokine Receptor-Like Factor 2 *(CRLF2)* gene [6]. *CRLF2* gene encodes the thymic stromal lymphopoetin receptor (TSLPR) and is located in the pseudoautosomal region 1 (PAR1) on the Xp22.3 and Yp11.3 chromosome [8]. During lymphopoiesis and inflammation, thymic stromal lymphopoietin (TSLP) binds to the heterodimer of TSLPR and the IL7RA α chain [8, 9]. *CRLF2* rearrangements include interstitial focal deletions juxtaposing *CRLF2* with the promoter region of the G-protein purinergic receptor (P2RY8) [8, 9], or translocation of *CRLF2* to the immunoglobulin heavy chain locus (IGH α) on 14q32.3 [6]. In both cases, *CRLF2* expression is enhanced, which increases activation of the downstream JAK-STAT signaling pathway that promotes proliferation of leukemic cells [10, 11]. In some cases, activating mutations in the *CRLF2* or *IL7RA* genes promote CRLF2 overexpression [8, 9].

The prognostic relevance of genomic aberrations in the *CRLF2* gene or *CRLF2* mRNA overexpression in pediatric BCP-ALL is unclear because of variations in methodologies, inclusion criteria and treatment protocols used by different studies [12–16]. Bugarin *et al.* demonstrated that high surface CRLF2 expression correlates with increased CRFL2 transcript levels in the leukemia cells isolated from CRLF2-overexpressing BCP-ALL patients [17]. However, the prognostic relevance of CRLF2 protein expression in pediatric BCP-ALL patients has not been reported. Moreover, the minimal residual disease (MRD) level in BCP-ALL patients with high CRLF2 transcript levels is controversial. Therefore, in this study, we prospectively analyzed bone marrow samples from 386 pediatric BCP-ALL patients at diagnosis and at days 15 and 33 of the treatment according to ALL-IC BFM 2009 protocol to determine the association of CRLF2 protein expression on the surface of leukemic cells with blast clearance in the bone marrow and treatment outcomes.

## RESULTS

### Basic characterization of pediatric BCP-ALL patients

**Figure 1:**
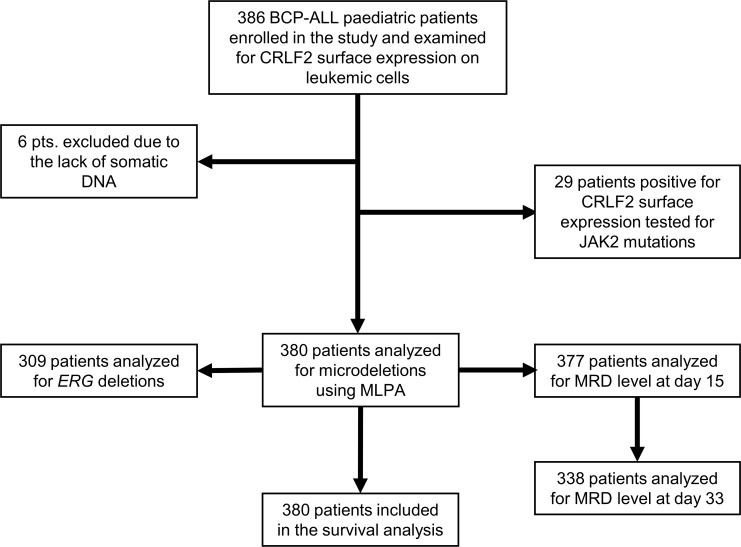
Flowchart shows selection strategy and classification of pediatric BCP-ALL patients at diagnosis and at days 15 and 33 of the treatment

Figure 1 describes the selection strategy employed in this study. Flow cytometry analysis at diagnosis showed that 29 out of 386 BCP-ALL patients (7.51%) expressed CRLF2 (TSLPR) protein on the surface of the leukemic cells. Table 1 summarizes the clinical and biological features of all CRLF2-positive (CRLF2pos; 29/386) and CRLF2-negative (CRLF2neg; 357/386) BCP-ALL patients at diagnosis and the level of minimal residual disease at days 15 and 33 of the treatment. In comparison to CRLF2neg patients, higher number of CRLF2pos patients were associated with Down syndrome (4/29 vs. 3/357, p=0.002) and *IKZF1* deletions (10/29 vs. 55/357, p=0.02). However, both CRLF2pos and CRLF2neg BCP-ALL patient groups showed similar distribution of age, sex, initial WBC count, and risk group characteristics (Table 1).

**Table 1:** Clinical and biological characteristics of pediatric BCP-ALL patients based on CRLF2 expression status

Variable	CRLF2neg n=357	CRLF2pos n=29	P value
**Median age (years)**^b^	4.3 [2.7-7.4]	4.3 [3.0-7.0]	0.68
**Males**^a^	190 (53)	17 (59)	0.71
**WBC count (10^9^/L)**^b^	10.3 [4.0-28.7]	8.5 [4.9-23.7]	0.91
**Poor steroid response**^a^	34 (10)	3 (10)	0.77
**Down syndrome**^a^	3 (2)	4 (36)	**0. 002**
**Hiperdiploidy**^a^	76 (23)	4 (13)	0.39
**Hipodiploidy**^a^	2 (0.6)	0 (0)	0.99
***MLL* rearrangements**^a^	18 (5.1)	1(3.4)	0.96
***ETV6-RUNX1* fusion**^a^	41 (15)	2 (6.8)	0.39
***BCR-ABL1* fusion**^a^	9 (2.5)	0 (0)	0.97
***ERG* deletion**^a^	13 (4.5)	1 (3.4)	0.98
***IKZF1* deletion**^a^	55 (16)	10 (35)	**0.02**
**Median MRD15 (%)**^b^	0.45 [0.05-3.50]	0.01 [0.001-0.42]	**0.001**
**Median MRD33 (%) (range)**^b^	≤0.001 [0.001-40.80]	≤0.001 [0.001-26.20]	0.827

^a^ Shown as number of cases, with percentages in brackets.

^b^ Shown as median value with interquartile range shown in square brackets.

### CRLF2 surface expression patterns BCP-ALL patients at diagnosis

**Figure 2:**
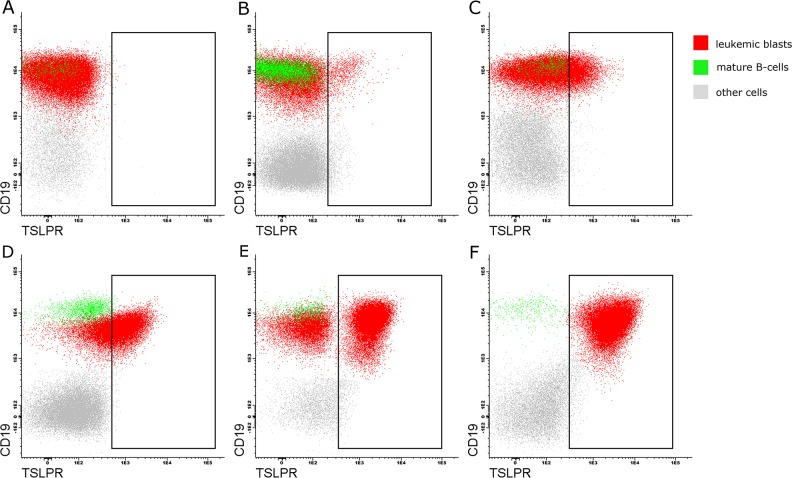
Flow cytometry profiles showing different CRLF2 expression patterns on leukemic blasts in the pediatric BCP-ALL patients Representative FACS plots showing surface CRLF2 staining in **(A)** negative control sample and CRLF2pos patients with **(B)** bimodal dim pattern (low expression on the blast subclone); **(C)** heterogeneous low expression (negative to dim) in some blasts; **(D)** homogeneous dim expression on all blasts; **(E)** bimodal strong pattern (high expression on the blasts subclone); and **(F)** homogeneous strong expression on all blasts. Note: The rectangles represent blasts positive for CRLF2; red - leukemic blasts; green - mature B-cells (CRLF2-negative), gray - other cells (CRLF2-negative).

CRLF2 expression patterns in CRLF2pos patients are shown in Table 2 and Figure 2. We observed 5 distinct patterns of CRLF2 expression in the CRLF2pos patients at diagnosis. These include [1] strong homogeneous expression of CRLF2 on leukemic blasts (n=6; nMFI-scores of 6-10; 99-100% positive); weak/dim homogeneous expression of CRLF2 (n=6; nMFI-scores of 2-5; 87.3-100% positive); [3] bimodal dim expression of CRLF2 on leukemic blasts suggesting the presence of a subclone (n=9; nMFI-scores of 1–2; 5.3–39.5% positive); [4] bimodal high expression of CRLF2 (n=1; nMFI-score of 6; 76% positive); and [5] low heterogeneous (negative-to-dim) expression of CRLF2 (n=7; nMFI-score of 1; 5.0–41.3% positive.

**Table 2:** Detailed characteristics of CRLF2 expression and accompanying genetic aberrations on leukemic blasts and negative reference cells (mature B-cells)

Sample ID	% positivity	MFI	nMFI-score	CRLF2 expression mode	Genetic aberrations
**1330**	36.9	112.1	1	neg-to-dim	Hiperdiploidy
**0959**	27.4	114.8	1	neg-to-dim	*ETV6-RUNX1*
**1288**	32.9	117.9	1	neg-to-dim	t(4;11)
**1584**	5.0	121.9	1	neg-to-dim	Hiperdiploidy
**1020**	41.3	137.7	1	neg-to-dim	*IKZF1*del
**1704**	6.0	138.7	1	neg-to-dim	*IKZF1*del, *JAK2*mut
**1089**	18.4	163.9	1	neg-to-dim	*P2RY8-CRLF2*
**1322**	9.0	272.41	1	bimodal dim	
**1443**	6.1	295.0	1	bimodal dim	*P2RY8-CRLF2, IKZF1*del
**981**	7.6	299.4	1	bimodal dim	Hiperdiploidy*, JAK2*mut
**1266**	5.5	316.2	1	bimodal dim	*IKZF1*del*, JAK2*mut
**1647**	5.3	404.0	2	bimodal dim	*ETV6-RUNX1*
**1460**	9.2	408.5	2	bimodal dim	*IKZF1*del, *ERG*del, Hiperdiploidy
**1571**	6.5	416.2	2	bimodal dim	
**0985**	39.5	562.2	2	bimodal dim	
**974**	14.4	579.9	2	bimodal dim	*IKZF1*del
**0973**	88.2	491.6	2	dim	*P2RY8-CRLF2, IKZF1*del
**1708**	87.3	672.4	2	dim	*P2RY8-CRLF2*
**1061**	100.0	883.3	3	dim	*P2RY8-CRLF2, IKZF1*del, *JAK2*mut
**1438**	97.8	916.0	3	dim	*P2RY8-CRLF2, JAK2*mut
**1431**	99.2	992.3	3	dim	*P2RY8-CRLF2*
**1497**	98.9	1503.1	5	dim	*P2RY8-CRLF2*
**1522**	76.0	1928.8	6	bimodal strong	*P2RY8-CRLF2, IKZF1*del, *JAK2*mut
**1048**	100.0	1780.5	6	strong	*P2RY8-CRLF2*
**1032**	99.4	862.7	6	strong	*P2RY8-CRLF2, IKZF1*del
**1459**	100.0	2217.0	7	strong	*CRLF2-IGH*, *IKZF1*del
**0982**	99.5	2519.7	8	strong	*P2RY8-CRLF2, IKZF1*del, *JAK2*mut
**1604**	100.0	3115.7	10	strong	*P2RY8-CRLF2*
**1331**	100.0	3408.4	10	strong	
negative control (mature B-cells)	0.0	43.55 (26.11-71.69)	0	neg	

Note: neg, negative; neg>mod, low expression in some blasts (heterogeneous expression from low to moderate); MFI interquartile range is reported for negative control (mature B-cells).

### Cytogenetic and microdeletion profiles of BCP-ALL patients

Primary chromosomal abnormalities were rare in leukemic cells from CRLF2pos BCP-ALL patients based on cytogenetic analysis. Cytogenetic analysis identified CRLF2pos patients with *ETV6-RUNX1* fusion (2/29), high hyperdiploid karyotype (4/29) and *MLL-ALF4* t (4; 11) gene arrangement (1/29; Table 2). These abnormalities were observed in BCP-ALL samples showing low heterogeneous (negative-to-dim) expression of CRLF2.

We screened 380 out of 386 BCP-ALL patients (98%) for the most common microdeletions using the MLPA method. Supplementary Table 2 shows the distribution of copy number abnormalities in CRLF2pos and CRLF2neg patients. Among the 29 CRLF2pos patients, 10 showed *IKZF1* deletions; 5 of the 10 with *IKZF1* deletions also showed *P2RY8-CRLF2* fusions. *PAX5* and *CDKN2A/B* microdeletions were observed in 8 and 9 CRLF2pos patients, respectively (Supplementary Table 1). *BTG1* deletions were more prevalent in CRLF2pos (3/29) than the CRLF2neg cases (23/353). However, the statistical data was insignificant. Since concurrent *ERG* and *IKZF1* deletions are prognostically significant in ALL patients [18], we analyzed 309 ALL samples (309/386; 80%) for the presence of *ERG* deletions. We observed *ERG* deletions in 14 cases (14/309; 4.4%), of which only one patient showed high CRLF2 expression (Supplementary Table 2).

We detected *P2RY8-CRLF2* fusion in all 13 CRLF2pos cases harboring the PAR1 deletion and 11 of these 13 cases showed dim or strong homogeneous surface expression of CRLF2. Since *JAK2* point mutations are associated with CRLF2 mRNA overexpression and treatment response in ALL patients [13], we sequenced the fragment of the *JAK2* gene that encodes the kinase and pseudokinase domains and identified *JAK2* mutations in 7 CRLF2pos samples. Among these, five samples showed *JAK2* mutations within the sequence encoding the pseudokinase domain (R683G) and two samples showed mutations in the kinase domain (T875A and T875N). Moreover, four samples with *JAK2* mutations showed *P2RY8-CRLF2* fusion. These four samples showed homogeneous dim or strong expression of CRLF2. None of the 29 CRLF2pos patients showed any point mutations in the *CRLF2* gene.

### Association of CRLF2 expression and MRD status at day 15 and 33

**Figure 3:**
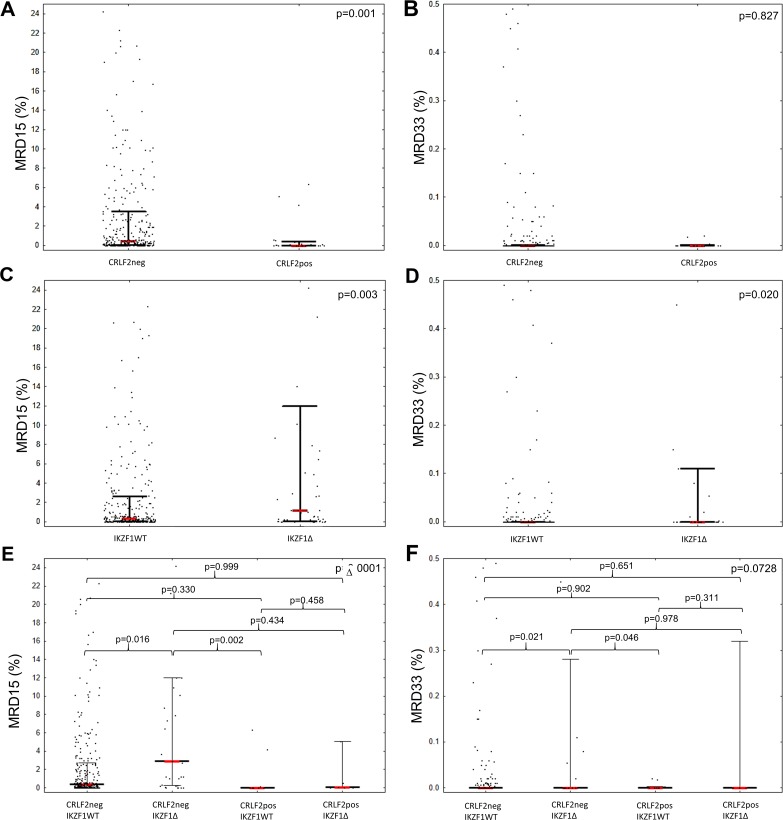
Comparison of MRD levels at **(A, C, E)** day 15 and **(B, D, F)** day 33 in patients with (A-B) surface CRLF2 expression, (C-D) *IKZF1* deletion and (E-F) CRLF2pos patients with *IKZF1* deletion.

We prospectively analyzed the MRD status at days 15 (MRD15) and 33 (MRD33) of the treatment in 377 and 338 patients, respectively. The probability of 5-year relapse-free survival (RFS) in the study cohort based on MRD15 status is presented in Supplementary Figure 1. CRLF2pos patients showed lower MRD15 levels than CRLF2neg patients (0.01% vs. 0.45%, p=0.001). However, the median MRD33 level was similar for both CRLF2pos and CRLF2neg patients [MRD33 CRLF2pos ≤0.001% (range 0.001-26.20%); MRD33 CRLF2neg ≤0.001% (range 0.001-40.80%); p=0.827]. In contrast, patients with *IKZF1* deletion showed higher median MRD15 and MRD33 levels than patients without *IKZF1* deletion [MRD15: IKZF1Δ=1.18%(0.06-12.00%) vs. IKZF1WT=0.33%(0.03-2.60%), p=0.003; MRD33: IKZF1Δ=0.001% (0.001-32.00%) vs. IKZF1WT=0.001% (0.001-40.80%), p=0.02]. Moreover, as shown in Figure 3, subgroup analysis showed that MRD15 level was lowest in CRLF2pos patients without *IKZF1* deletion (IKZF1WT/CRLF2pos: 0.001% [0.001-0.01%]), higher in CRLF2pos with *IKZF1* deletion (*IKZF1*Δ/CRLF2pos: 0.10% [0.02-5.06%]) as well as CRLF2neg without *IKZF1* deletion (*IKZF1WT*/CRLF2neg: 0.40% [0.04-2.70%]), and highest in CRLF2neg patients with *IKZF1* deletion (*IKZF1*Δ/CRLF2neg: 2.90% [0.25-12.0%], p=0.0001). Multivariate analysis confirmed the mitigating effect of CRLF2 overexpression in patients with *IKZF1* deletions (Supplementary Table 3). The CRLF2/*IKZF1* status significantly affected the MRD15 and MRD33 levels in BCP-ALL patients after adjusting for age at diagnosis and initial WBC count. Patients with CRLF2pos/IKZF1WT genotype showed lowest MRD15 and MRD33 levels, whereas patients with CRLF2neg/IKZF1Δ showed highest MRD15 and MRD33 levels than the other groups (p=0.003). CRLF2pos patients with *IKZF1* deletion and CRLF2neg patients with *IKZF1* deletion showed similar MRD15 and MRD33 levels (p=0.95). MRD15 level correlated with the percentage of blasts positive for CRLF2 surface expression at diagnosis. Patients with higher percentage of CRLF2 positive blasts at diagnosis showed lower MRD15 (R=-0.43, p<0.05; Supplementary Figure 2).

### Prognostic significance of CRLF2 expression at diagnosis

**Figure 4:**
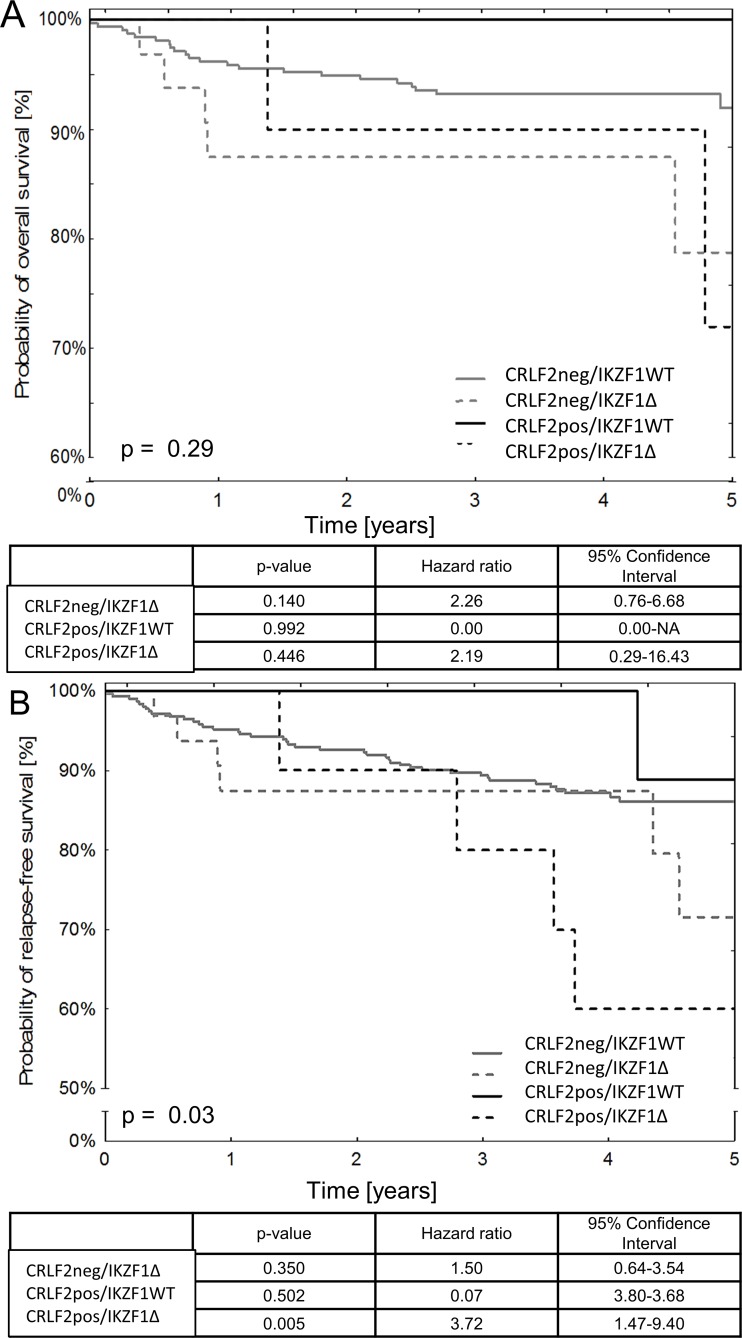
Survival outcomes in pediatric BCP-ALL patients based on the status of CRLF2 expression and *IKZF1* deletions The Kaplan-Meier survival curves show **(A)** overall survival (OS) and **(B)** relapse-free survival (RFS) of pediatric BCP-ALL patients based on the status of surface CRLF2 protein expression and *IKZF1* deletions. Also included are tables showing survival estimates based on the Cox proportional hazard regression model for different categories of patients.

The median follow-up time for the study subjects was 4.09 (3.56-4.78) years. Univariate analysis showed that there was no correlation between CRLF2 expression status and overall survival (OS) or relapse-free survival (RFS). OS and RFS rates in CRLF2pos and CRLF2neg BCP-ALL patients were similar (OS: 88% vs. 90%; p=0.73; RFS: 71% vs. 85%; p=0.48). Overall, we noted six adverse events in CRLF2pos patients. These included five bone marrow relapses and one death due to complications in a patient with Down syndrome. Among these, three patients belonged to the intermediate risk group and the remaining three belonged to the high-risk group. Moreover, two patients that died due to relapse showed *JAK2* mutations, whereas, five out of six relapse patients showed *IKZF1* deletions. Two patients with relapse showed high MRD15 level that exceeded 10%. Furthermore, patients with *IKZF1* deletions showed lower OS and RFS rates than those without *IKZF1* deletions (OS: 72% vs. 94%, p=0.01; RFS: 62% vs. 87%, p=0.008). Therefore, we re-analyzed survival rates for patients with different combinations of *CRLF2 and IKZF1* genetic alterations and observed that the 5-year OS rates were similar for all subgroups (Figure 4). However, IKZF1Δ/CRLF2pos patients showed lower RFS rates than IKZ1WT/CRLF2neg patients (Figure 4). We also analyzed if *P2RY8-CRLF2* fusion correlated with patient survival. Our results showed no significant differences in OS and RFS between the *P2RY8-CRLF2pos* and *P2RY8-CRLF2neg* groups (RFS: 84% vs. 88%, p=0.42; OS: 92% vs. 90%, p=0.90; Figure 4).

## DISCUSSION

High *CRLF2* mRNA expression and *IKZF1* deletions were associated with low survival rates in pediatric BCP-ALL [6]. However, prognostic relevance of *CRLF2* aberrations in pediatric ALL patients is controversial. CRLF2 overexpression activates the JAK/STAT pathway and therefore represents a potential target for JAK inhibitors in cancer therapy [10, 11]. However, in contrast to *IKZF1* deletions, the prognostic value of *CRLF2* aberrations and/or CRLF2 mRNA high expression is not clear. Moreover, prognostic significance of CRLF2 protein expression on leukemic cells has not been studied previously. Therefore, we investigated the clinical significance of surface CRLF2 protein expression in pediatric ALL patients at diagnosis as well as early response to the induction therapy at days 15 and 33. We also assessed the survival outcomes in ALL patients treated according to the BFM 2009 protocol, based on surface CRLF2 expression status with or without *IKZF1* deletions.

We detected surface expression of CRLF2 in 8% of BCP-ALL patients analyzed. These findings corroborate previous BCP-ALL studies (AIEOP-BFM 2000, St Jude and COG, DCOG ALL-10) that reported high CRLF2 mRNA expression in 5-7% ALL patients [12–15]. However, this number of ALL cases with high CRLF2 mRNA expression based on the presumed cut-off values for positivity in different methodological approaches; nearly 17% of ALL cases showed high CRLF2 mRNA expression in studies that used highly sensitive qualitative and quantitative PCR based methods [19]. Bugarin *et al.* reported that ~10% of pediatric BCP-ALL patients exhibited surface CRLF2 expression at diagnosis by performing flow cytometry analysis using a cutoff value of 1% positivity [17]. However, cutoff values below 2% in flow cytometry analysis can result in false positives because of non-specific staining, especially in the bone marrow samples analyzed ≥24 h after isolation [20].

We observed different patterns of surface CRLF2 expression in CRLF2pos BCP-ALL patients. Among the 386 BCP-ALL patients, 12 (3.1%) showed dim or strong homogeneous expression of CRLF2 (nMFI-scores ranging from 2 to 10), whereas, 10 (2.6%) exhibited bimodal dim or strong CRLF2 expression, thereby suggesting the presence of CRLF2-positive subclones. Bugarin *et al.* also reported CRLF2-positive subclones in 2/421 (0.5%) patients [17]. In our study, the size of subclones ranged from 5.3 to 14.4% of blast cells in 10/11 (91%) patients with bimodal CRLF2 expression. This was consistent with previous findings, which showed that leukemic clones with *CRLF2* aberrations were not predominant at diagnosis and relapse [19].

The deletion in the pseudoautosomal region 1 (PAR1 region) results in *P2RY8-CRLF2* fusion and represents the most common molecular event associated with CRLF2 overexpression in BCP-ALL patients [8]. Among the 29 CRLF2pos patients in our study cohort, 44% showed the incidence of *P2RY8-CRLF2* fusion. Furthermore, half of the *P2RY8-CRLF2* fusion-positive CRLF2pos cases showed simultaneous CRLF2 protein expression. This suggested the presence of the *P2RY8-CRLF2* fusion in minor subclones of the leukemic cells. This hypothesis is supported by the high concordance between *P2RY8-CRLF2* clone size and the corresponding high CRLF2 mRNA expression in BCP-ALL patients [19].

In previous reports, late relapse results in poor outcomes among the pediatric BCP-ALL patients, especially those stratified in the intermediate risk group associated with high CRLF2 mRNA expression and *CRLF2* rearrangements (for example, *P2RY8-CRLF2* fusion) [15, 16]. This suggests potential clinical value in evaluating surface CRLF2 expression by flow cytometry to predict treatment failure in pediatric BCP-ALL patients. In our study cohort, *P2RY8-CRLF2* fusion did not significantly influence survival. However, CRLF2pos patients with *IKZF1* deletion were associated with increased risk of relapse.

We determined MRD15 and MRD33 levels to investigate if initial response to treatment contributed to leukemia recurrence in CRLF2pos/IKZF1Δ patients by analyzing blast clearance in the bone marrow of CRFL2pos patients with or without *IKZF1* deletions. Previous studies have shown that BCP-ALL patients with high CRLF2 expression are more sensitive to treatment as measured by MRD levels [14, 21]. In contrast, HR-ALL cases with high CRLF2 mRNA expression from the COG cohort are associated with higher MRD levels and poor RFS at the end of induction therapy [13]. Patients harboring *IKZF1* deletions alone show slower blast clearance in the bone marrow during induction therapy than the patients with the wild type *IKZF1* gene [13, 14]. In our study group, CRLF2pos patients at diagnosis were associated with low MRD15 levels. Conversely, the MRD15 level was high in patients with *IKZF1* deletions and low in CRLF2pos patients with *IKZF1* deletions (IKZF1Δ/CRLF2pos). This finding corroborates a previous report by Van der Veer *et al.*, which showed increased risk of relapse in high *CRLF2* expressing patients with *IKZF1* deletions [14]. Vesely C. *et al.* also reported that *IKZF1* deletion was the only relapse-predicting molecular indicator in *P2RY8-CRLF2* positive ALL cases [22]. Since we did not assess surface CRLF2 expression during follow-up studies, we could not analyze its relationship with *IKZF1* deletions at the follow-up time points.

In conclusion, our study demonstrates that surface CRLF2 expression in pediatric BCP-ALL patients harboring *IKZF1* deletions are associated with increased risk of relapse.

## MATERIALS AND METHODS

### Patient characteristics

We prospectively enrolled 386 newly diagnosed BCP-ALL children (median age: 4.3 [2.7-7.2] y) in this study. Patients were diagnosed and treated according to the ALL IC BFM 2009 protocol between August 2011 and March 2014 in the clinical centers of the Polish Pediatric Leukemia/Lymphoma Study Group: Department of Pediatrics, Hematology, Oncology and Diabetology, Medical University of Lodz, Department of Pediatric Hematology and Oncology, Medical University of Silesia, Zabrze, Department of Pediatrics, Oncology and Hematology, Medical University of Warsaw, Department of Pediatric Hematology, Oncology, Transplantology, Medical University of Poznan, Department of Pediatric Hematology and Oncology, Medical University of Lublin, Department of Pediatric Hematology and Oncology, Children's Hospital in Olsztyn, Department of Pediatrics, Hematology, Oncology and Endocrinology, Medical University of Gdansk, Department of Transplantology, Pediatric Oncology and Hematology, Medical University of Wroclaw, Department of Pediatric Oncology, Medical University, Bialystok, Department of Pediatrics, Medical University of Silesia, Katowice, Regional Specialistic Pediatric Hospital, Kielce, Department of Pediatric Hematology and Oncology, Collegium Medicum in Bydgoszcz, Mikolaj Kopernik University, Department of Pediatrics, Hematology and Oncology, Pomeranian Medical University. Patients with T cell and mature B cell ALL were not included in the study. Detailed description of the risk group assignment is presented in the online Supplementary Materials and Methods. The research protocol was approved by the Ethics Committee at the Medical University of Lodz and informed consent was obtained from all participants and/or their parents.

### Flow cytometric assessment of CRLF2 and minimal residual disease detection in BCP-ALL

Bone marrow samples from all BCP-ALL patients were analyzed by 8-color flow cytometry protocol subscribed by the EuroFlow Consortium [23] at the one center (Department of Pediatric Hematology and Oncology, Medical University of Silesia) in Zabrze. In addition to the EuroFlow panel for BCP-ALL [24], we added an additional tube containing a combination of TSLPR-PE (clone 1D3, Biolegend, San Diego, CA, USA), CD19-PE-Cy7 (Beckman Coulter, Brea, CA, USA), CD34-PerCP-Cy5.5 (Becton Dickinson, San Diego, CA, USA) and CD45-Pacific Orange (Life Technologies, Carlsbad, USA) antibodies. The bone marrow samples collected at diagnosis of ALL as well as at days 15 and 33 of the therapy, were incubated with 1x FACS Lyse (Becton Dickinson) for erythrocyte lysis and the cells were stained and analyzed with the FACS Canto II flow cytometer (Becton Dickinson) within 1-48 h of sample collection. At least 50,000 stained cells were acquired by FACS. The reproducibility of the results was ensured by complying with the standard operating protocols developed by EuroFlow [24] and daily quality assessment of the flow cytometer with fluorescent beads (Sphero Rainbow Calibration Particles; Spherotech, Lake Forest, IL, USA) [23]. The data was analyzed using the FACS Diva 6.1 software (Becton Dickinson).

Moreover, 377 patients on day 15 and 338 patients on day 33 were subjected to flow cytometry analysis to determine the minimal residual disease (MRD) levels after induction treatment. Bone marrow samples were subjected to bulk lysis in accordance with guidelines of the EuroFlow consortium and stained with the following cocktail of monoclonal antibodies: CD22-PE, CD34-PerCP-Cy5.5, CD10-APC, CD38-APC-H7 (Becton Dickinson, San Diego, CA, USA), CD19-PE-Cy7 (Beckman Coulter, Brea, CA, USA), CD20 (Biolegend, San Diego, CA, USA) and CD45-Pacific Orange (Life Technologies, Carlsbad, USA) antibodies. In addition, cells were stained with the nucleus staining dye, Syto-16 (Life Technologies, Carlsbad, USA) to exclude dead cells and cell debris [24, 25]. The CRLF2 (TSLPR) expression on blast cells was determined by assessing [1] the percentage of blasts positive for CRLF2 with the cutoff set at 5%-positivity beyond the negative control level of mature B-cells, and [2] the normalized median fluorescence intensity (nMFI). The maximal MFI value for CRLF2-stained leukemic blasts was normalized by subtracting the median MFI of the negative reference population (mature B-cells) and dividing the positively stained population into 10 equal nMFI-scores, according to a protocol reported by Sedek et al. [26]. CRLF2 expression scores of 1–5 were considered negative-to-dim for low heterogeneous expression or dim for homogeneous expression. CRLF2 expression was considered as strongly homogenous for nMFI scores ≥ 6. In cases with bimodal CRLF2 expression with at least 5% positive blasts, the subclone was ascribed an nMFI score that corresponded to either dim or strong bimodal groups. Representative CRLF2 expression profiles are presented in Figure 2. CRLF2 antigen was not assessed during follow-up studies.

### Multiplex Ligation-Dependent Probe Amplification (MLPA)

Targeted copy number screening of eight selected loci was performed by multiplex ligation-dependent probe amplification (MLPA) using both P202-B1 and P335-B2 SALSA MLPA kits (MRC-Holland, Amsterdam, The Netherlands). Detailed MLPA protocol, DNA and RNA isolation protocol, multiplex-PCR testing of *ERG* deletions, detection of the *P2RY8-CRLF2* fusion transcript, direct sequencing of *CRLF2* and *JAK2* genes and the statistical analyses are described in the *online Materials and Methods Supplementary File.*

## SUPPLEMENTARY MATERIALS FIGURES AND TABLES


