# Supplementary material for: Surface expression of Cytokine Receptor-Like Factor 2 increases risk of relapse in pediatric acute lymphoblastic leukemia patients harboring IKZF1 deletions
Source: Oncotarget. 2018 May 25;9(40):25971–82. doi: 10.18632/oncotarget.25411 (PMC5995260; doi:10.18632/oncotarget.25411)
Supplement: Supplementary file 1 [file oncotarget-09-25971-s001.pdf]

Surface expression of Cytokine Receptor-Like Factor 2 increases risk of relapse in pediatric acute lymphoblastic leukemia patients harboring *IKZF1* deletions

SUPPLEMENTARY MATERIALS

Minimal residual disease monitoring

Bone marrow samples were processed according to lyse/wash protocol, with 1x FACS Lyse used for erythrocyte lysis (Becton Dickinson) and analysed with FACS Canto II flow cytometer (Becton Dickinson). To ensure the sensitivity level of (at least) 10^{-4}, low-cellular samples, were first subjected to erythrocyte lysis in 1x ammonium chloride solution (Pharm Lyse, Becton Dickinson) in a so-called bulk lysis protocol. Subsequently, the suspension of leukocytes was stained in a single 8-color tube with antibodies adequate for the blasts' phenotype at diagnosis. CRLF2 expression was not assessed during follow-up studies.

DNA and RNA isolation

Genomic DNA from the leukemic blasts, was isolated from previously Ficoll-gradient separated bone marrow samples using the FlexiGene DNA Kit (Qiagen, Valencia, USA), according to the manufacturer's protocol. Total RNA from 1×10^7 of leukemic cells resuspended in TRIzol solution was extracted using chloroform extraction according to the Invitrogen protocol. Nucleic acid purity and concentration was assessed on NanoDrop Spectrophotometer-8000 (Thermo Fisher Scientific, Walthman, USA).

Multiplex Ligation-Dependent Probe Amplification (MLPA)

Targeted copy number screening of eight selected loci was performed in the cohort using multiplex ligation-dependent probe amplification (MLPA) using both the P202-B1 and P335-B2 SALSA MLPA kits (MRC-Holland, Amsterdam, The Netherlands). Both assays include probes for each of the eight exons of the *IKZF1* gene and are able to detect deletions of the whole gene as well as all types of focal intragenic deletions. Selected exons of the genes *BTG1, CDKN2A/B, EBF1, ETV6, IKZF1, PAX5, RB1, IGH* and the PAR1 region (located approximately 230 kbp downstream of *SHOX, CRLF2, CSF2RA* and *IL3RA*) are also covered. Probe mix and hybridization buffer (MRC-Holland) were added in equal amounts to 120 ng of genomic DNA followed by heat denaturation and overnight hybridization of gene-specific probes at 60°C. Subsequently, ligation was performed and the ligation products were amplified by PCR using a 6-FAM fluorophore-labelled primer set (MRC-Holland). The amplification products were quantified and identified by capillary electrophoresis on an ABI 3130 DNA analyser (Life Technologies, Carlsbad, USA). Data were analysed using Gene Marker v.5.4 software (Softgenetics, State College, USA). Normalization of the data was carried out by dividing the peak area of each probe by the average area corresponding peak of control probes. This normalized peak pattern was divided by the average peak pattern of all the samples in the same experiment. The resulting values were 1 for every wild-type peak, 0.5 for heterozygous deletions and 1.5 for heterozygous duplications.

Detection of the P2RY8-CRLF2 fusion transcript

For those samples showing PAR1 deletion in MLPA, we confirmed the presence P2RY8-CRLF2 fusion on mRNA level. First strand cDNA synthesis was carried out on 1 μg of RNA using High-Capacity cDNA Reverse Transcription Kit (Life Technologies). The presence of P2RY8-CRLF2 chimeric transcript caused by the fusion of the first noncoding exon of *P2RY8* and the first coding exon of *CRLF2* was identified by RT-PCR with primers derived from both genes. The sequences of designed primers are listed in Supplementary Table I. Subsequently the product was sequenced using Sanger's method with a BigDye Terminator Cycle Sequencing kit v. 3.1 (Life Technologies) on an ABI 3130 DNA analyser (Life Technologies) following the manufacturer's instructions.

Analysis of ERG deletions

The presence of *ERG* deletions was tested using multiplex-PCR based method. We followed conditions and primers sequences which were published by Zaliova M et al [18]. In each positive case, after the verification of the presence of PCR product by electrophoresis on a 2% agarose gel and visualization under UV light, amplicons were purified using QIAquick PCR Purification Kit (Qiagen, Valencia, USA). Direct DNA sequencing using Sanger's method was performed with a BigDye Terminator Cycle Sequencing kit v. 3.1 (Life Technologies) on an ABI 3130 DNA analyser (Life Technologies) following the manufacturer's instructions. Resulting sequences were analysed using Sequencher software (Gene Codes Corporation, Ann Arbor, USA).

Direct sequencing for detection of point mutations in CRLF2 and JAK2 genes

To search for *CRLF2 and JAK2* point mutations, direct sequencing was performed on DNA from leukemic cells with *CRLF2* overexpression detected by flow cytometry. Primers for PCR reactions were designed in all coding regions of *CRLF2* and *JAK genes*. The sequences of designed primers are listed in Supplementary Table 4. Amplification was carried out with an initial denaturation step at 95 ºC for 5 min, followed by 30 cycles of denaturation at 95 ºC for 30 s, annealing at 60 ºC for 30 s, extension at 72 ºC for 60 s, and a final extension step at 72 ºC for 10 min. After the verification of PCR products by electrophoresis on a 2% agarose gel and visualization under UV light amplicons were purified using QIAquick PCR Purification Kit (Qiagen, Valencia, USA). Direct DNA sequencing using Sanger's method was performed with a BigDye Terminator Cycle Sequencing kit v. 3.1 (Life Technologies) on an ABI 3130 DNA analyser (Life Technologies) following the manufacturer's instructions. Resulting sequences were analysed using Sequencher software (Gene Codes Corporation, Ann Arbor, USA) and aligned against reference sequences obtained from UCSC Genome Bioinformatics (NM_022148.3 and NM_004972) (University of California, Santa Cruz, CA, USA).

Statistical analysis

Differences in categorical variables were evaluated using Chi^2 test or two-tailed Fisher's exact tests. For continuous variables the Mann-Whitney's U test or Kruskall-Wallis nonparametric analysis of variance were used depending on the number of compared groups. Post-hoc comparisons were performed using the Dunn's test if significance was ascertained in Kruskal-Wallis test. Overall survival (OS) was defined from diagnosis to last follow-up, with censoring of alive patients at last follow-up. Relapse-free survival (RFS) was defined as the time from diagnosis to relapse. Estimated RFS and OS analyses were compared using the log-rank test. Kaplan-Meier survival curves were used to represent probabilities of survival over time. Multivariate analysis of survival was performed using Cox' proportional hazard regression models. Multivariate analysis of MRD was performed using analysis of covariance (ANCOVA) after $\log_{10}$ of MRD values. All analyses were performed in Statistica Software version 12.5 PL (StatSoft, Tulsa, USA). P values <0.05 were considered statistically significant.

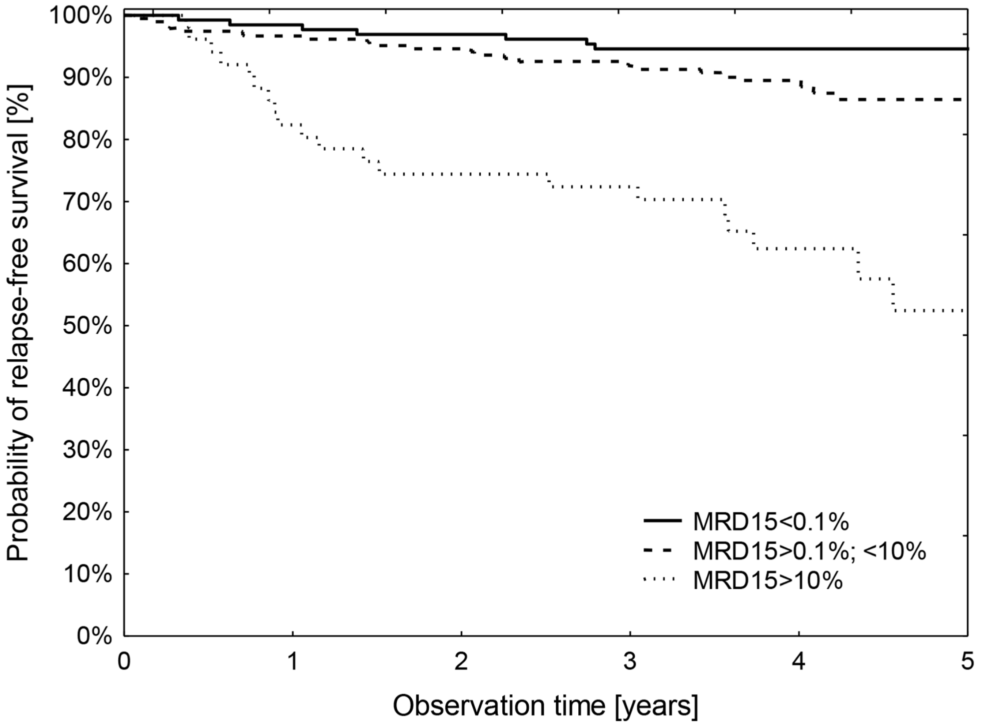

Variable	Group	P-value	Hazard ratio (HR)	95% CI
MRD15	0.1 to 10	0.29	2.16	(0.96-4.83)
	>10	10^{-7}	8.69	(3.8-19.84)

Supplementary Figure 1: Probability of relapse-free survival (RFS) with respect to MRD level at day 15 in the study cohort.

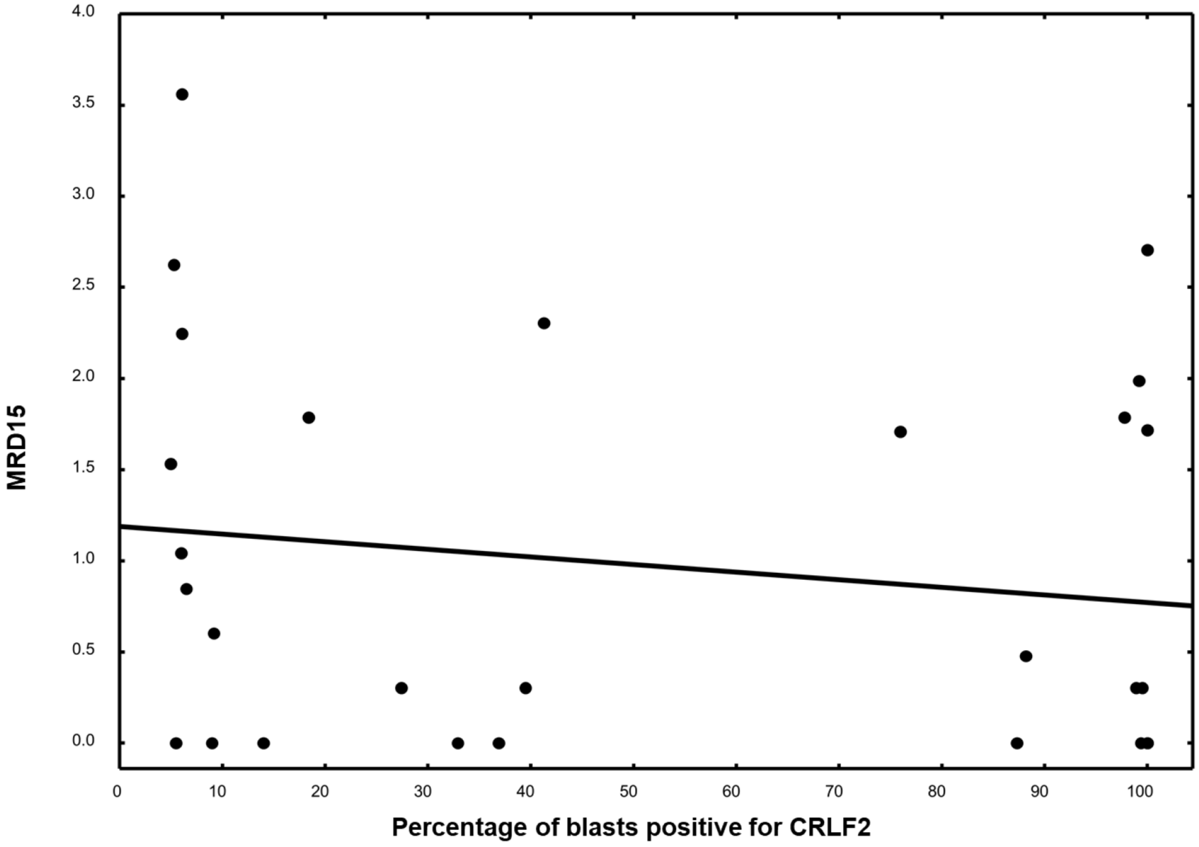

Supplementary Figure 2: Correlation between the level of minimal residual disease at day 15 and percentage of blasts positive for CRLF2 in CRLF2pos ALL samples at diagnosis of leukemia (R=-0.43, p<0.05).

Supplementary Table 1: Copy number abnormalities according to CRLF2 expression

	N. of cases	*IKZF1*	*PAX5*	*CDKN2A/B*	*BTG1*	*EBF1*	*ETV6*	*RB1*	*PAR1*
**CRLF2pos**	29	10 (35%)	8 (28%)	9 (31%)	3 (10%)	1 (3%)	5 (17%)	2 (7%)	13 (44%)
**CRLF2neg**	353	55 (16%)	57 (16%)	83 (24%)	23 (7%)	9 (3%)	77 (22%)	19 (5%)	15 (4%)

Supplementary Table 2: Number of patients positive for the specific genetic aberrations with respect to the pattern of CRLF2 expression

CRLF2 expression mode	nMFI range	*IKZF1* del	*ERG*del	*JAK2*mut	*P2RY8-CRLF2*
**Strong (n=6)**	6-10	3	0	1	4
**Bimodal strong (n=1)**	6	1	0	1	1
**Dim (n=6)**	2-5	2	0	2	6
**Bimodal dim (n=9)**	1-2	4	1	2	1
**Neg-to-dim (n=7)**	1	2	0	1	1

Supplementary Table 3: Multivariate ANCOVA model for MRD at day 15. Regression parameters refer to $\log_{10}$ MRD15

Variables	P value	Beta
**Age at diagnosis**	0.003	0.155
**WBC log**	<0.001	0.295
**IKZF1Δ/CRLF2pos**	0.95	0.007
**IKZF1WT/CRLF2pos**	0.003	-0.320
**IKZF1Δ/CRLF2neg**	0.002	0.318

Supplementary Table 4: Sequences of *CRLF2* and *JAK2* primers used in direct sequencing and sequences of primers detecting *CRLF2-P2RY8* fusion on cDNA

Primers	Sequences
F1_JAK2	GGAGATTTCAAAACCGAGTAGAGCC
R1_JAK2	TAGCCCCACAAGAATGTATCCTCAG
F2_JAK2	GGCTTACACAGGGGTTTCCTCAGA
R2_JAK2	CTCTATTGTTTGGGCATTGTAACCTTCT
F3_JAK2	TTGTTTAGACTCCTACTCTTGCTGTTGT
R3_JAK2	ATTACAGATTTATTTTTGTTTCCAGGT
F4_JAK2	TAAAATGTAAAACAGGAAGCATAGGAGTC
R4_JAK2	AGTAGTAAAGATGAACAAACACAAATCAAAGT
F5_JAK2	CACATCCAACCCCTCCAAAATAAAG
R5_JAK2	TCAGTCAAGGAGATTAGAATATGTATAAAGCA
F6_JAK2	CTATTTGAGTTTCCCTGTATCATTTAGTATTT
R6_JAK2	TAACACAGCATTTCTCCAACATCTACA
F7_JAK2	GGGTGACAGAGCGAGACTTC
R7_JAK2	CCTCTGGGCATTGGCATAAG
F8_JAK2	CAGTTTATTTTGGTTTGCCTGAAGAG
R8_JAK2	GATTTCCTAATGTCTACTTCAACACGGT
F9_JAK2	AACCGTGTTGAAGTAGACATTAGGAAA
R9_JAK2	CGTGGACTATAACCATGACTATAAGACCT
F10_JAK2	GCCTGCTGTGATAACTCTTTTCTCTAC
R10_JAK2	GGGTGAGCCATTGGTGGAGTAG
F11_JAK2	TTGAAAACATACAAAAGCACACATATACTAAA
R11_JAK2	CTGTAAATCTACTTTGGTCTCAGAATGAAGG
F_CRLF2-P2RY8	GGACAGATGGAACTGGAAGGG
R_CRLF2-P2RY8	CCTCCTTGCCCCAAAGCCATC
F1_CRLF2	GCTTCCTGCCTGTAATCATTTCCC
R1_CRLF2	AACCTCCATGCTCATTGTTTAAGTTTTAC
F2_CRLF2	GCCTCACCGTGAACTTCATGC-
R2_CRLF2	GACAGTGAGTCAGCCATCCCTTG
F3_CRLF2	GCTTTTTTGTTTTGTTGCTGAATGGTC
R3_CRLF2	ATGAGATTGCTTTTCGGTACTTACGGTAAT
F4_CRLF2	GAGAGTTCCTTCCTTCACAAACCTACAGG
R4_CRLF2	GAGCGAGGTCTGATGAAACAGCG
F5_CRLF2	TGGACAGACAGGTTACATCACTAATCAT
R5_CRLF2	GACAGCCTCCCTCCCACCTCC
F6_CRLF2	TGAGGGAGACTGGTTAGGGATGAG
R6_CRLF2	CAGGGAAATGACTCTATCAGGCG
